# Evaluation of expression of common genes in the intestine and peripheral blood mononuclear cells (PBMC) associated with celiac disease 

**Published:** 2020

**Authors:** Ensieh Khalkhal, Fatemeh Nobakht, Mohammad Hossain Haidari, Zahra Razaghi, Mahsa Ghasemzad, Melika Sheikhan, Mohammad Rostami Nejad

**Affiliations:** 1 *Proteomics Research Center, Faculty of Paramedical Sciences, Shahid Beheshti University of Medical Sciences, Tehran, Iran*; 2 *Chemical Injuries Research Center, Systems Biology and Poisoning Institute, Baqiyatallah University of Medical Sciences, Tehran, Iran*; 3 *Laser Application in Medical Sciences Research Center, Shahid Beheshti University of Medical Sciences, Tehran, Iran*; 4 *Gastroenterology and Liver Diseases Research Center, Research Institute for Gastroenterology and Liver Diseases, Shahid Beheshti University of Medical Sciences, Tehran, Iran*; 5 *Basic and Molecular Epidemiology of Gastrointestinal Disorders Research Center, Research Institute for Gastroenterology and Liver Diseases, Shahid Beheshti University of Medical Sciences, Tehran, Iran*

**Keywords:** Celiac Disease, PBMC, Intestinal Tissue, Autoimmune, Inflammatory

## Abstract

**Aim:**

this study was conducted to investigate expression of the genes associated with CD in the target tissue in order to estimate contribution of each single gene to development of immune response. Then, the same set of genes was evaluated in peripheral blood mononuclear cells (PBMCs).

**Background:**

Celiac disease (CD) is a chronic systemic autoimmune disease of the small intestine occurring in genetically-susceptible individuals. There are several genes related to immune response.

**Methods:**

For this purpose, the genes related to CD were extracted from public databases (documents of proteomics and microarray-based techniques) and were organized in a protein-protein interaction network using the search tool for retrieval of interacting genes/proteins (STRING) database as a plugin of Cytoscape software version 3.6.0. The main genes were introduced and enriched via ClueGO to find the related biochemical pathways. The network was analyzed, and the most important genes were introduced based on central indices.

**Results:**

Among 20 CD genes as hub and bottleneck nodes, there were 7 genes with common expression in blood and intestinal tissue (C-X-C motif chemokine 11(CXCL11), granzyme B (GZMB), interleukin 15(IL-15), interleukin 17(IL-17A), interleukin 23(IL-23A), t-box transcription factor 21(TBX21), and tumor necrosis factor alpha-induced protein 3(TNFAIP3)).

**Conclusion:**

The enriched biological process related to the central nodes of celiac network indicated that most of hub-bottleneck genes are the well-known ones involved in different types of autoimmune and inflammatory diseases.

## Introduction

 Celiac disease (CD) is a chronic systemic autoimmune disease of the small intestine occurring in genetically-susceptible individuals (1). CD as a multifactorial disease involves genetic elements (human leukocyte antigen (HLA)-DQ2 and HLA-DQ8) and environmental trigger (gluten) (2).

The primary mechanism involved in development of CD is related to an inappropriate adaptive immune response to gliadin, a prolamin found in wheat and related cereals. Prolamins contain critical epitopes presented by either HLA-DQ2 or HLA-DQ8, leading to induction of a cluster of differentiation 4(CD4)+ T-lymphocytes response(3).

High levels of pro-inflammatory cytokines are produced by the activated CD4+ T-lymphocytes (4). Cytokines can prime the innate immune response by polarizing dendritic cells and intraepithelial lymphocyte function (5). Following innate immune response, intestinal cells can be directly exposed to intestinal damage. Intestinal damage is developed gradually from completely normal mucosa, to mucosal inflammation, followed by crypt hyperplasia, and culminating in villous atrophy (6). 

Gene expression profiling (GEP) as a robust test is potentially used for classification purposes, with a high inter laboratory reproducibility (7, 8).Gene expression studies are a useful approach to provide evidence regarding implication of several functional pathways in complex diseases, such as CD (9). Genes displaying differential expression in CD intestine and PBMC compared to normal and common expression of both CD intestine and PBMC can be useful in developing a gene expression-based diagnostic test. Moreover, histological and functional alterations associated with CD may be revealed by analyzing the genes differentially expressed in CD (10, 11) , or by monitoring the genes potentially involved in intestinal immune response in CD (12). 

There are several genes related to immune response. Therefore, this study was performed to investigate expression of the genes associated with CD in the target tissue in order to estimate contribution of each single gene to development of immune response. Then, the same set of genes was evaluated in PBMC or whole blood. 

## Methods

Microarray experiments were used in CD intestine tissues and PBMC or whole blood was measured in the microarray-based techniques. Whole genome profile with differential expression in CD small intestine compared to control (533 genes), and in PBMC or whole blood compared to control (252 genes) was collected from public databases and gene expression omnibus(GEO) dataset published by March 2019. Also, all the genes with differential expression were collected. Fold change (FC)≥2 was considered to screen the differentially expressed genes. The protein-protein interaction (PPI) network was constructed using the search tool for retrieval of interacting genes/proteins (STRING) database as a plugin of Cytoscape software version 3.6.0 (13). Such that, genes (proteins) were nodes, and edges between gene nodes were formed when a gene was found to be significantly differentially expressed in the disease state. The main connected component of PPI network was analyzed by the network analyzer plugin of Cytoscape software. The most important topological properties of the PPI network nodes including degree and betweenness of nodes were considered to rank nodes of the network. The number of top 20% of the genes based on degree values was selected as hub genes and 20% of genes based on betweenness were identified as bottleneck nodes. The top 20 nodes based on degree value and betweenness centrality were selected as hub and bottleneck nodes, respectively. Interactions between hub-bottleneck nodes were identified by a related sun-network. Hub-bottleneck nodes of the celiac network were enriched by Kyoto encyclopedia of genes and genomes(KEGG) via ClueGO (14, 15). The resulting biochemical pathways were clustered. A p-value less than 0.05 was considered as statistically significant. 

## Results

**Figure 1:**
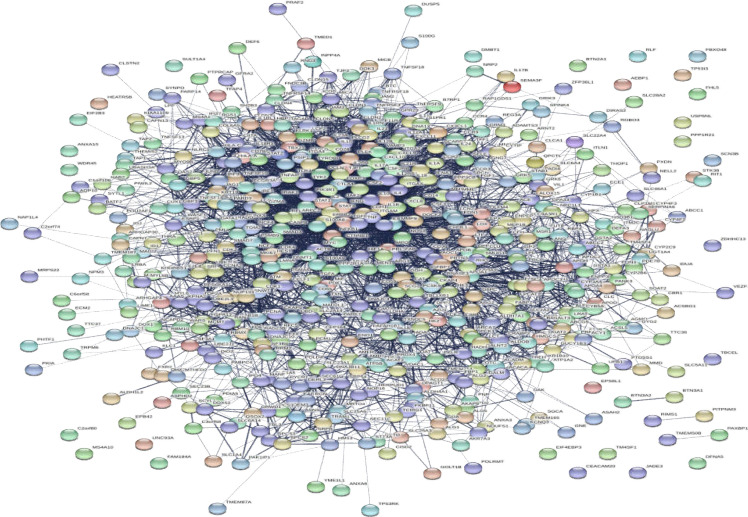
PPI network of CD constructed by 533 genes extracted from STRING database

**Figure 2:**
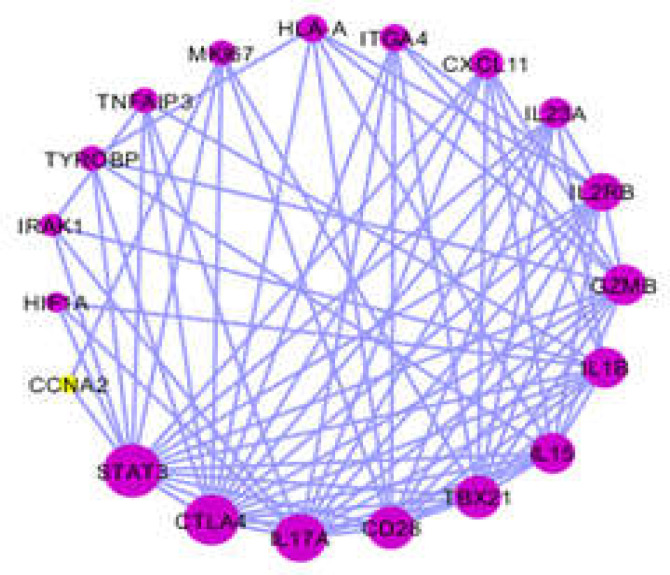
The 20 central nodes of CD network organized in a sub-network

For integrating the data provided through an experimental study, literature survey, or database, differential expressions of the genes present in the patients with CD were combined compared to those of healthy controls. There are 533 genes with differential expression in CD small intestine compared to control and 259 genes with differential expression in CD PBMC or whole blood vs. control (16-29). The genes related to CD were extracted from the STRING database. As shown in Figures 1, 2 a total of 533 genes were included in the main connected component. The network was analyzed, and the nodes were ranked on the basis of centrality parameters. Top 20% of nodes based on the degree value and betweenness centrality were selected and organized in 2 groups. As described in the Methodology Section, the top 20 nodes based on degree value and betweenness centrality were selected as hub and bottleneck nodes, respectively as presented in Table1. 

**Table 1:** The top 20 nodes based on degree value and betweenness centrality were selected as hub and bottleneck nodes with differential expression in CD blood and intestine compared to control. CXCL11, GZMB, IL-15, IL-17A, IL-23A, TBX21, and TNFAIP3 had common expression in CD blood and intestinal tissue

	Hub& bottleneck	Degree	Betweenness centrality	Expression	
				Intestine	Blood
1	APOA1	40	0.00634588	Down (20, 21)	-
2	CCNA2	30	0.00841483	Down (30)	-
3	CD28	63	0.00839965	Up (23)	-
4	CTLA4	88	0.02961893	Up (19)	-
5	CXCL11	45	0.02059243	Up (19(31))	Up (32)
6	GZMB	57	0.01230656	Up (19)	Up
7	HIF1A	35	0.00923055	Up (27)	-
8	HLA-A	40	0.00915393	Up (30)	-
9	IL-15	67	0.01475644	Up (19)	Up (33)
10	IL-17A	86	0.01610106	Up (19)	Up (28, 34)
11	IL-1B	95	0.01405327	Up (19)	-
12	IL-23A	35	0.0535164	Up (21)	Up (35)
13	IL-2RB	53	0.00721186	Up (14)	-
14	IRAK1	32	0.00518941	Down (18)	-
15	ITGA4	32	0.03352843	Down (18)	-
16	MKI67	32	0.01219633	Up (19)	-
17	STAT3	115	0.00616552	Up (21)	-
18	TBX21	55	0.02993936	Up (19)	Up (28, 34)
19	TNFAIP3	35	0.00532498	Up (22)	Up (23)
20	TYROBP	40	0.0078105	Up (19)	-

Also, change in expression of the genes in CD small intestine and PBMC or whole blood vs. control was investigated via the literature survey, and the findings are presented in Table1. Among 20 CD genes as hub and bottleneck nodes, there were 7 genes with common expression in blood and intestinal tissue (CXCL11 (C-X-C motif chemokine ligand 11), GZMB (granzyme B), IL-15 (interleukin 15), IL-17A (interleukin 17A), IL-23A (interleukin 23A), TBX21 (T-box 21), and TNFAIP3 (tumor necrosis factor alpha-induced protein 3)).

Since, the gene attribution in biological processes is an important role of gene in the investigated disease, 20 genes were enriched through gene ontology (GO) and significant processes were determined as shown in Table2. 

**Table 2:** Biological process of the genes as hub-bottleneck nodes

Associated genes	GOTerm
[CTLA4, TNFAIP3, TYROBP]	Negative regulation of B cell activation
[APOA1, IL-15, IL-1B, IL-2RB]	Positive regulation of phagocytosis
[IL-15, IL-2RB, STAT3]	Response to IL-15 IL-15-mediated signaling pathway Cellular response to IL-15
[CD28, IL-23A, TYROBP]	Production of IL-10 Regulation of IL-10 production Production of IL-10 Regulation of IL-10 production
[CD28, IL-1B, STAT3, TYROBP]	Positive regulation of cytokine biosynthetic process
[IL-1B, STAT3, TYROBP]	IL-6 biosynthetic process Regulation of IL-6 biosynthetic process Positive regulation of IL-6 biosynthetic process
[IL-23A, STAT3, TBX21]	CD4-positive, alpha-beta T cell differentiation involved in immune response T-helper cell lineage commitment T-helper 17 cell differentiation T-helper 17 type immune response T-helper 17 cell lineage commitment Activation of alpha-beta T cell involved in immune response
[CD28, IL-1B, TBX21, TNFAIP3]	Production of IL-2
[HLA-A, IL-23A, TYROBP]	Positive regulation of leukocyte-mediated cytotoxicity
[CD28, IL-23A, TYROBP]	
[CD28, IL-1B, TBX21, TNFAIP3]	Regulation of IL-2 production
[HLA-A, IL-1B, TBX21]	T cell cytokine production Regulation of T cell cytokine production
[CD28, HLA-A, IL-1B, IL-23A, TBX21]	
[IL-15, IL-1B, IL-23A, TNFAIP3]	Regulation of defense response to virus Regulation of defense response to virus by host
[CD28, HLA-A, IL-1B, IL-23A, TBX21]	Positive regulation of adaptive immune response Positive regulation of lymphocyte-mediated immunity Positive regulation of adaptive immune response based on somatic recombination of immune receptors built from immunoglobulin superfamily domains
[IL-15, IL-23A, TYROBP]	Regulation of natural killer cell activation Positive regulation of natural killer cell activation
[CD28, IL-1B, STAT3, TYROBP]	Positive regulation of cytokine biosynthetic process
[HLA-A, IL-1B, IL-23A, TBX21]	Regulation of T cell-mediated immunity
[CD28, HLA-A, IL-15, IL-23A]	Proliferation of alpha-beta T cell
[HLA-A, IL-1B, IL-23A]	Positive regulation of T cell-mediated immunity
[CD28, IL-15, IL-23A, STAT3, TBX21]	T cell selection
[IL-23A, STAT3, TBX21]	T cell lineage commitment
[CD28, HLA-A, IL-23A]	Regulation of alpha-beta T cell proliferation
[IL-23A, STAT3, TBX21]	Positive T cell selection Lineage commitment of alpha-beta T cell Lineage commitment of CD4-positive or CD8-positive, and alpha-beta T cell
[CD28, HLA-A, IL-23A]	Positive regulation of alpha-beta T cell proliferation
[IL-17A, IL-23A, TYROBP]	Positive regulation of osteoclast differentiation
[IL-23A, STAT3, TBX21]	Differentiation of alpha-beta T cell involved in immune response of CD4-positive, alpha-beta T cell lineage commitment

**Table 3:** The enriched pathways from KEGG related to 20 central nodes of CD network

Associated genes	GOTerm
[CD28, IL-15, ITGA4]	Intestinal immune network for IgA production
[IL-1B, IRAK1, ITGA4]	Leishmaniasis
[CD28, IL-1B, IL-2RB, IRAK1, STAT3, TNFAIP3]	Measles
[CD28, CTLA4, IL-15, IL-17A, IL-1B, IL-23A]	Rheumatoid arthritis
[HIF1A, IL-17A, IL-1B, IL-23A, IL-2RB, STAT3, TBX21]	Differentiation of Th17 cell
[IL-17A, IL-1B, IL-23A, STAT3, TBX21]	Inflammatory bowel disease (IBD)
[CD28, GZMB, HLA-A, IL-1B]	Type I diabetes mellitus Graft-versus-host disease
[CD28, CTLA4, GZMB, HLA-A]	Autoimmune thyroid disease
[CD28, GZMB, HLA-A]	Allograft rejection

**Table 4:** Biological process and enriched pathways from KEGG related to the genes as hub and bottleneck nodes with common expression in both CD small intestine biopsy and blood

Associated genes	GOTerm
[IL-15, IL-23A, TBX21]	T cell selection
[IL-15, IL-23A, TNFAIP3]	Regulation of defense response to virus by host
[IL-17A, IL-23A, TBX21]	Inflammatory bowel disease (IBD)

The enriched pathways from KEGG related to 20 hub and bottleneck nodes of CD network are also shown. Ten terms related to 20 hub- bottleneck nodes were identified and clustered (Table 3). Also, biological process and enriched pathways from KEGG related to the genes as hub and bottleneck nodes with common expression in both CD small intestine biopsy and blood were identified and clustered (Table 4). Important roles of these biological processes in relation to CD are discussed in detail in the following section.

## Discussion

There are large amount of data in genomics and proteomics (high throughput methods) applied as suitable screening tools (36). In this research, the reported data related to CD were screened by PPI network analysis to find the crucial genes among them. Differentially expressed genes present in the patients with CD compared to healthy controls were combined. There are 533 genes with differential expression in CD small intestine compared to control and 259 genes with differential expression in CD PBMC or whole blood vs. control. Totally, 533 genes were analyzed using the PPI network and 20 central genes were introduced as hub-bottleneck nodes. The roles of hub-bottleneck genes are introduced in Table 2. 

The GO can be useful for obtaining the information about roles of a gene set (37). The enriched biological process related to the central nodes of CD network (Table 3) indicated that most of hub-bottleneck genes are the well-known ones involved in different types of autoimmune and inflammatory diseases (38, 39). Among the mechanisms involved in innate immune system, chemokine signaling pathway is involved in development of CD. CXCL11 is a chemokine that binds to its receptor CXCR3 as a member of family G-protein. Expression of CXCR3 and its ligands is strongly associated with autoimmune diseases, such as CD (38). High expression levels of CXCR3, CXCL10, and CXCL11 in small intestinal biopsies of the untreated patients with CD suggest that these chemokines are able to induce an innate immune response and are important factors in development of CD (29, 38). 

Immune reaction in CD involves an adaptive innate immune response and is characterized by the presence of anti-gluten and anti-transglutaminase 2 antibodies, and expression of multiple cytokines and other signaling proteins in the intestinal epithelial membrane. Interleukins, such as IL-15 are a group of cytokines, which can prime innate immune response by polarizing dendritic cells and intraepithelial lymphocyte. They have been reported to be associated with numerous inflammatory conditions including CD, Crohns҆ disease, psoriasis, Rheumatoid arthritis, and type 1 diabetes (40-42). IL-15 contributes to pathogenesis of autoimmune diseases, such as CD through activation of immune responses. Therefore, genetic or environmental factors controlling expression of IL-15 and responsiveness in the intestine are likely involved in pathogenesis of CD (43). The roles of IL-15 and IL-23 in generation, activation ,and expansion of CD4+ T-lymphocytes still remain unknown (44). IL-15 provides activating and survival signals to indirectly modulate CD4+ T cell responses. Activated CD4+ T-lymphocytes are thought to perpetuate inflammation by secreting pro-inflammatory cytokines, such as interferon γ (IFNγ), IL-17, and interleukin 21(IL-21) that act on immune and non-immune cells. Regulation of T cell differentiation and cytokine pathway seems to be provided by cytoplasmic and nuclear transcription factors. TBX21 has been identified as a key transcription factor for development of T helper lymphocytes. The signal transducer and activator of transcription (STAT) proteins are cytoplasmic transcription factors activated via receptors from cytokines of signal transducer and activator of transcription 3(STAT3) that provides multiple functions in cytokine -mediated signaling in many cell types including differentiation, survival, apoptosis ,and mobility. It is involved in generation of cells and acute inflammatory response. STAT3 is activated by several cytokines(45, 46).

TNFAIP3 is involved in regulation of the nuclear factor kappa B (NF-kB) inflammatory signaling pathway in pathology of coeliac disease and is one of the key mediators in this nuclear activating complex (47). TNFAIP3 is an attractive candidate for both inflammatory and autoimmune pathogenesis. TNFAIP3 is required for mediation of the NF-kB signal by innate immune receptors via de-ubiquitination of several NF-kB signaling factors. NF-kB as a transcription complex has a key role in regulation of the cellular immune response to stimuli. In various inflammatory disorders including CD, arthritis ,and inflammatory bowel disease, NF-kB is activated and NF-kB pathway plays an independent role in innate mechanisms of disease development (48). 

These cytokines lead to histopathological changes like villous atrophy and crypt hyperplasia (49, 50). Upon cell death, mRNAs are released into the surrounding environment and then, reaching peripheral blood circulation or body fluids; hence detection of tissue-specific mRNA in bio fluids might be used as a biomarker for specific tissue damage and specific tissue event. In addition, a biomarker panel for CD introduced through analyzing and screening significantly differentially expressed genes should be considered as an important player in pathology of CD.
